# The HIV manifestations within the gastrointestinal tract: A pictorial review

**DOI:** 10.4102/sajr.v21i2.1233

**Published:** 2017-11-14

**Authors:** Alexandra Renn, Farhat Kazmi, Nasir Khan, Bhavin Rawal, Elaine O’Boyle

**Affiliations:** 1 Chelsea and Westminster Hospital, London, United Kingdom

## Abstract

The aim of the pictorial review are to review the HIV manifestations within the gastrointestinal tract. We have detailed five conditions, with reference to the patients’ CD4 count – gastrointestinal tuberculosis, Kaposi’s sarcoma, small bowel lymphoma, cytomegalovirus colitis and anal carcinoma.

## Introduction

### HIV, AIDS and CD4 count

The acquired immunodeficiency syndrome (AIDS) was first described as a clinical entity in 1981, and HIV (human immunodeficiency virus) was identified as the causative organism in 1983.^1^ The HIV virus is recognised by host cells that exhibit the CD4 surface glycoprotein. In a healthy person, their CD4 count ranges from 500 cells/mm^3^ to 1200 cells/mm^3^.^2^ The HIV virus replicates within these cells, resulting in their destruction and defective T cell homeostasis and subsequent immunodeficiency. Hence, AIDS is a disorder of cell-mediated immunity, clinically defined by the presence of multiple opportunistic infections and certain characteristic malignancies.^1,2^

### HIV within the gastrointestinal tract

Gastrointestinal pathology in HIV includes a number of rare infections and tumours related to immunosuppression. The gastrointestinal disease manifestations of HIV can be subdivided into two categories: Infections and HIV acquired neoplasm.

**Infections:**

- Cytomegalovirus
- Tuberculosis

**HIV acquired neoplasm:**

- Kaposi sarcoma
- Non-Hodgkin’s lymphoma
- Squamous cell carcinoma

The presentation of each disease is variable, the incidence increasing should the patients’ CD4 count fall below certain thresholds, as detailed below^3,4,5,6,7^:

- Kaposi sarcoma: < 350 cells/μL^3^
- Anal carcinoma: < 350 cells/μL^4^
- Non-Hodgkin’s lymphoma: < 200 cells/μL^5^
- Tuberculosis: < 200 cells/μL^6^
- Cytomegalovirus colitis: < 100 cells/μL^7^

## Clinical findings

We have pictorially reviewed the various gastrointestinal manifestations in HIV, with reference to patients CD4 count.

### Small bowel lymphoma

We have focused specifically on small bowel lymphoma. The incidence of primary small bowel lymphoma is rare and is estimated to attribute to just 0.9% of all gastrointestinal tract tumours.^8^ There is a slight male predominance, with a male: female ratio of 3:2.6.^8^

However, the incidence is increasing, primarily because of the increased rate of HIV. One study^9^ has stated that over half of the patients diagnosed with small bowel lymphoma (specifically, non-Hodgkin’s Lymphoma) also have AIDS.

Small bowel lymphoma typically involves the terminal ileum, and becomes less frequent proximally.^10^

The radiological features of small bowel lymphoma include:

- Focal thickening of the bowel wall, measuring between 1 and 7 cm^9^
- Fungating masses
- Tumour infiltration of the myenteric nerve plexus, resulting in aneurysmal bowel dilatation^8^
- Solid mass lesion (rare).^8^

**FIGURE 1:**
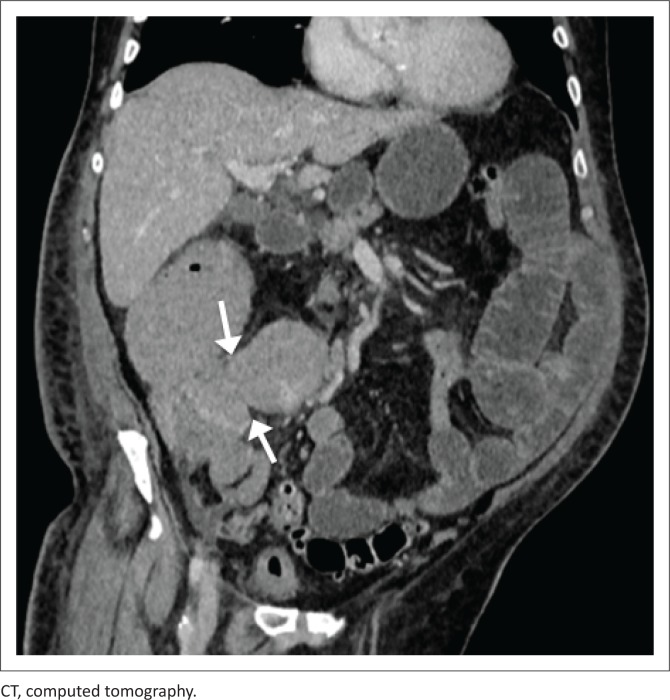
Coronal reconstruction of the portovenous phase of a CT abdomen of a 50-year-old male patient demonstrating lymphomatous circumferential thickening of terminal ileum and caecum (arrowed).

**FIGURE 2:**
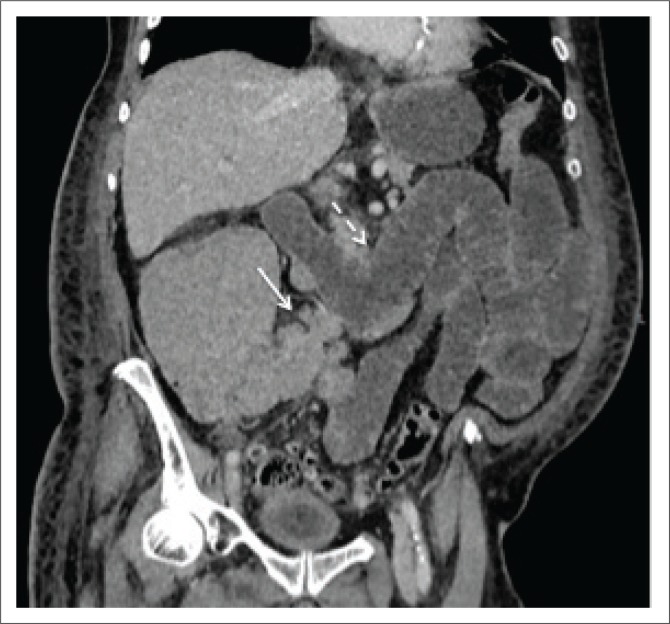
Coronal reconstruction of portovenous phase enhancement of a 50-year-old male (as Figure 1), demonstrating enlarged lymph nodes along the ileocaecal vessels (solid arrow) and upstream fluid-filled loops of small bowel (dashed arrow).

As demonstrated in Figures 1 and 2, there is lymphomatous infiltration of the ileocaecal valve, with circumferential thickening of the terminal ileum and caecum, lymphadenopathy and upstream small bowel dilatation. The patient was a 50-year-old male, with a CD4 count of 190 (cells/μL). Subsequent histological analysis following endoscopic biopsy confirmed the diagnosis of non-Hodgkin’s lymphoma.

### Cytomegalovirus colitis

Cytomegalovirus (CMV) is a prevalent type of herpes simplex virus. CMV colitis is common, with an incidence of 5% – 10%, typically affecting the severely immunocompromised HIV patient.^7^

The radiological features of CMV colitis are non-specific, and include bowel wall thickening, mucosal ulceration and luminal narrowing. It can be either diffuse or segmental, and typically involves the ascending colon and caecum, but can also extend to the terminal ileum. Unsurprisingly, CMV colitis is often misdiagnosed as inflammatory bowel disease.^7,11^ However, Murray et al.^11^ identified there to be relative sparing of the transverse colon, helping distinguish the two. Also, unlike inflammatory bowel disease, 42% of those infected also have ascites.^11^ Although the features are non-specific, diagnosis should be considered in HIV patients with a CD4 count of less than 100 cells/μL.^7,11^

**FIGURE 3:**
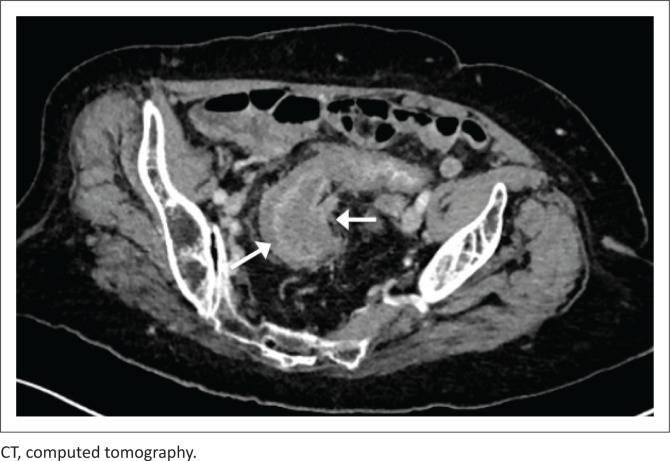
Axial reconstruction of portovenous phase–enhanced CT, demonstrating circumferential mural thickening of the sigmoid colon (arrowed).

**FIGURE 4:**
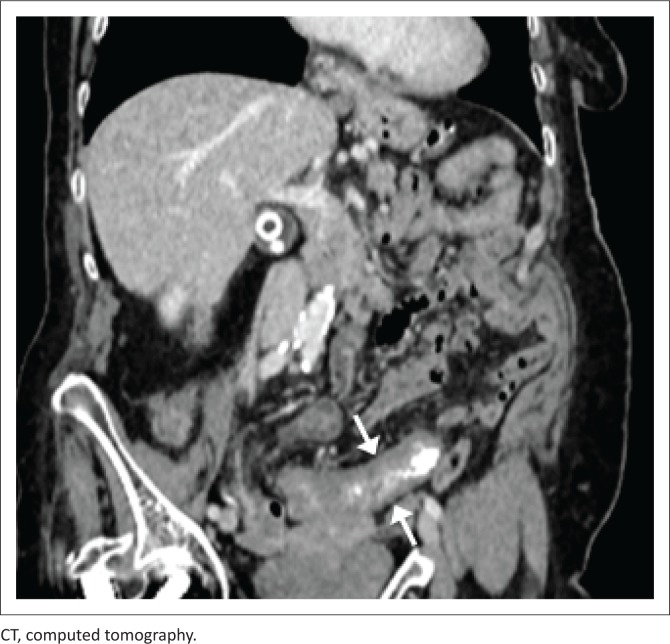
Coronal reconstruction of portovenous phase–enhanced CT, of the above patient, again demonstrating circumferential mural thickening of the sigmoid colon.

Figures 3 and 4 demonstrate mural thickening and mucosal enhancement of the sigmoid, in a male patient with an undetectable CD4 count. This was the only segment of bowel affected. A diagnosis of CMV colitis was made upon serology.

### Anal squamous cell carcinoma

The main causative agent of anal squamous cell carcinoma is infection with the human papilloma virus (HPV). Although HIV is not a direct cause, it is an indicator of further co-infection of sexual transmitted diseases, particularly in patients engaging in anoreceptive intercourse.^12^ Furthermore, patients with HIV are seven times more likely to have persistent HPV.^4^

MRI is the preferred imaging modality for the assessment of anal tumours, providing detailed information regarding size, location and local invasion. The malignant tissue within the anal canal demonstrates low signal intensity on T1-weighted imaging. On T2-weighted imaging and short tau inversion recovery (STIR) sequences, it appears as intermediate signal intensity, lower than ischioanal fat.^13^

On CT, anal squamous cell carcinoma appears as a solid, enhancing mass, becoming more heterogeneous as its size increases^12,13^

**FIGURE 5:**
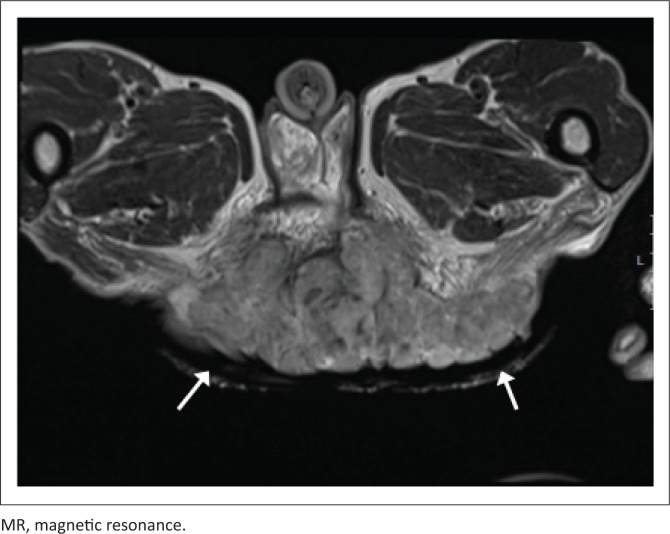
T2-weighted MR imaging, axial slice of a 42-year-old male patient, demonstrating a large anal soft tissue mass (arrowed) of intermediate signal intensity, with local soft tissue invasion. Histology confirmed the diagnosis of squamous cell carcinoma.

**FIGURE 6:**
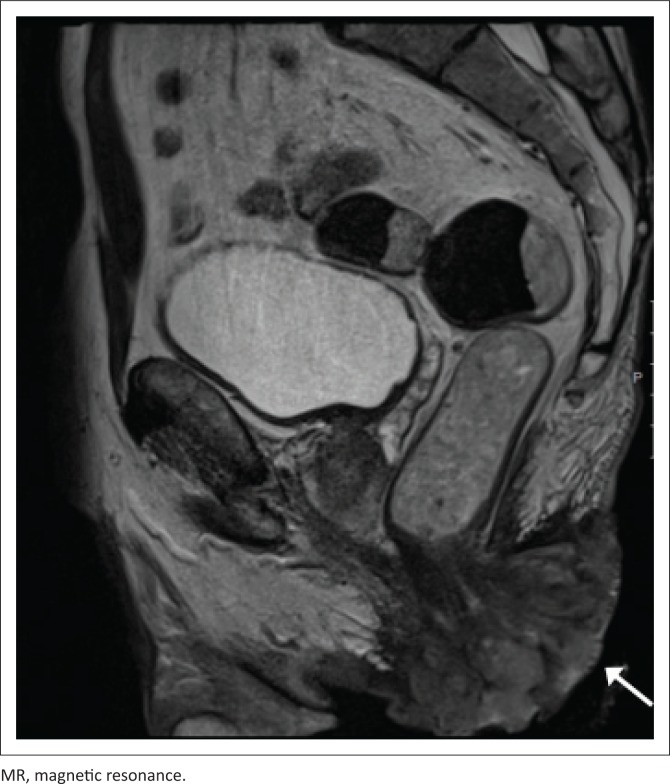
Sagittal T2-weighted MR imaging of a 42-year-old male patient (as shown in Figure 5), demonstrating a large anal soft tissue mass (arrowed) of intermediate signal intensity, with local soft tissue invasion.

Figures 5 and 6 demonstrate a 42-year-old HIV-positive male patient, with a CD4 count of 16 cells/μL. T2-weighted imaging indicates a large, soft tissue mass, resulting in extensive soft tissue invasion. The mass demonstrates intermediate signal intensity, lower than the surrounding adipose tissue.

### Gastrointestinal tuberculosis

Worldwide, tuberculosis (TB) is prevalent, particularly amongst patients with HIV, and it has been estimated that up to 70% of patients will develop TB in their lifetime.^6^ As detailed above, the chance of developing TB greatly increases with a fall in CD4 count, with the threshold being approximately 200 cells/μL.^6^ Gastrointestinal manifestations are typically secondary to pulmonary TB.^14^

Abdominal tuberculosis can affect any segment of the gastrointestinal tract but most commonly the terminal ileum^8^ because of the large volume of lymphoid tissue in this area.^14^ On CT/MRI, this will appear as circumferential thickening of the affected segment of the bowel, with surrounding lymphadenopathy.^14^

However, CT/MRI features are often non-specific, and can be confused with inflammatory bowel disease or malignancy.^14^ Characteristic appearances include:

- Asymmetric thickening of the terminal ileum and medial wall of the caecum^14^
- Significant lymphadenopathy, with central areas of reduced attenuation.^14^

**FIGURE 7:**
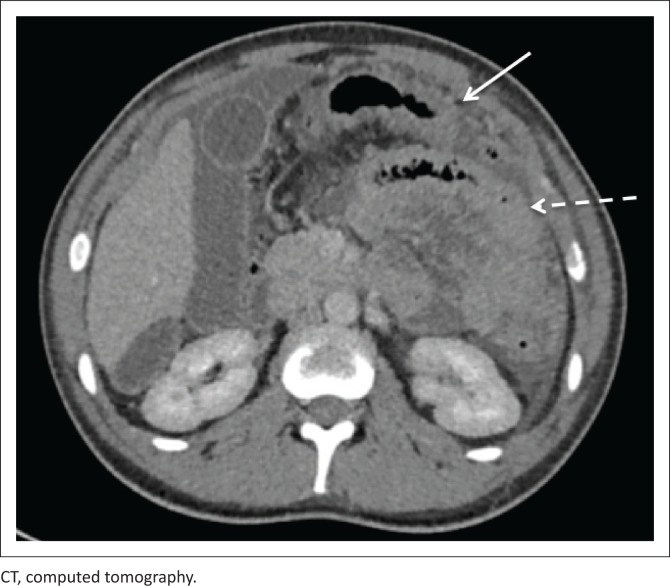
Axial reconstruction of portovenous phase–enhanced CT in a 23-year-old male patient with a CD4 count of 70 cells/μL. The image demonstrates diffuse serosal thickening of the jejunum (dashed arrow), ascites and peritoneal nodularity (solid arrow). Histological diagnosis of TB was made following ascitic tap.

**FIGURE 8:**
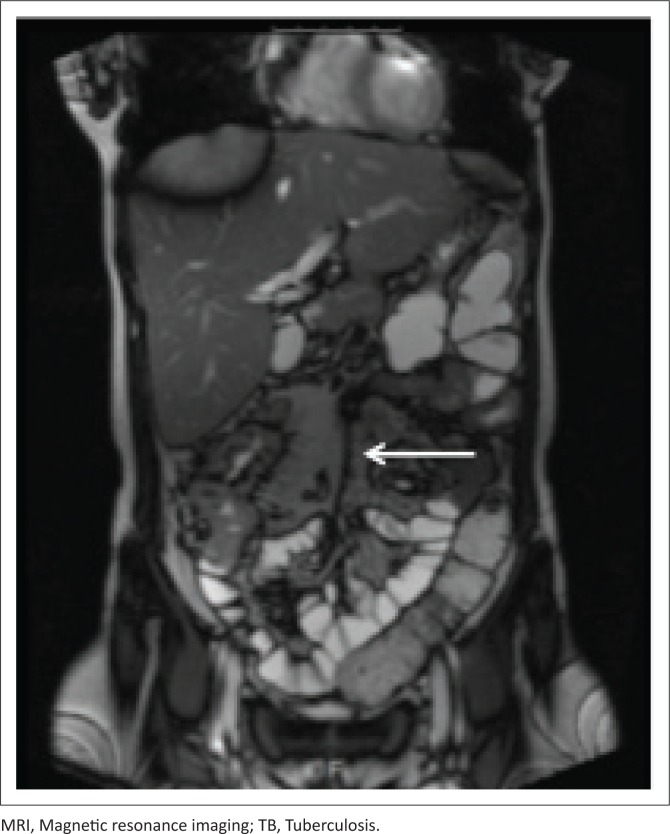
Coronal T2-weighted MRI. This image is of a 30-year-old male with a CD4 count of < 100 cells/μL, demonstrating a 4 cm segment of thickened distal and terminal ileum, with localised perforation. Confirmation of TB was made upon endoscopic biopsy and histology.

Figures 7 and 8 demonstrate the imaging findings of two different patients with abdominal TB. Both patients’ CD4 count at presentation was < 100 cells/μL.

### Kaposi sarcoma

Kaposi sarcoma (KS) is considered to be an AIDS defining illness. Gastrointestinal KS is the most common involvement in disseminated disease, being identified in around half of the patients.^1,15^ Involvement includes any part of the gastrointestinal tract, including the gallbladder. The most commonly affected site is the duodenum.^15^

Portovenous-enhanced CT is the preferred imaging modality, with 80% of patients with disseminated disease demonstrating enhancing lymph nodes.^15^ The masses most commonly appear polypoid (< 3 cm), although larger masses are possible.

**FIGURE 9:**
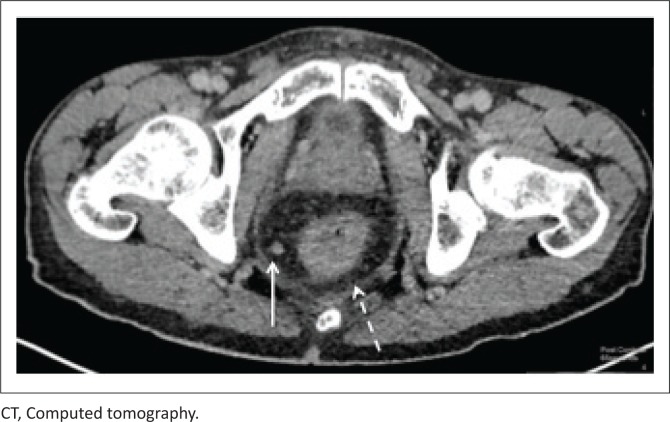
Axial reconstruction, portovenous-enhanced CT imaging of a 50-year-old male patient. The image demonstrates rectal thickening (dashed arrow) and an enhancing mesorectal node (solid arrow).

**FIGURE 10:**
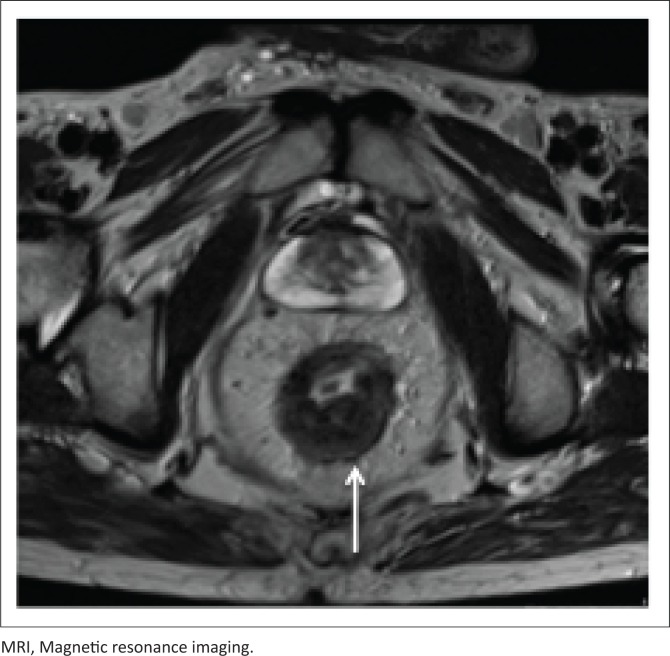
Axial T2-weighted MRI imaging. The patient is a 50-year-old male with thickening of the posterior wall of the rectum. MRI was performed to aid surgical management.

Figures 9 and 10 demonstrate a 50-year-old male patient, with CD4 count of 320 cells/μL at initial presentation, with rectal thickening and an enhancing mesorectal node. The diagnosis of rectal KS was made upon transrectal biopsy.

## Conclusion

The gastrointestinal tract is a common location for many AIDS defining and non-defining illnesses. As detailed above, depletion of the patients CD4 count increases their likelihood of developing certain pathologies. In conditions such as CMV colitis and abdominal TB, the imaging features can be non-specific, with few subtle defining characteristics. An understanding of the disease processes associated with HIV, together with correlation with the patients CD4 count, aids diagnosis and management.
